# What really matters to older adults in life and treatment: a qualitative study on the role of connectedness in person-centered care

**DOI:** 10.1186/s12877-026-07736-9

**Published:** 2026-06-03

**Authors:** Anneke G. Julien, Willeke M. Ravensbergen-Roobol, Veerle M. G. T. H. van der Klei, Mabel J. E. Maissan, Bas F. M. van Raaij, Frederiek van den Bos, Simon P. Mooijaart, Jacobijn Gussekloo, Yvonne M. Drewes

**Affiliations:** 1 Department of Internal Medicine, Section Gerontology and Geriatrics, Leiden University Medical Center, Leiden, The Netherlands; 2 LUMC Center for Health and Aging (LUCHA), Leiden University Medical Center, Leiden, the Netherlands; 3 Department of Public Health and Primary Care, Leiden University Medical Center, Leiden, The Netherlands

**Keywords:** Connectedness to life, Finiteness of life, Treatment goals, Shared decision making, Relationality

## Abstract

**Background:**

Healthcare professionals experience challenges in eliciting what really matters to older adults in person-centered care with shared decision-making. The framework of internal and external connectedness to life is a promising model for explicating individual values and preferences. We aimed to explore the relation between this framework and what really matters to older adults in life and treatment.

**Methods:**

This qualitative study followed a thematic content analysis of 25 interviews with heterogeneous older adults aged ≥ 70 years in the Netherlands.

**Results:**

Our analyses identified four themes. First, participants (mean age, 80 years, range 70–99 years) considered feeling connected to life to play a vital role in life, disease and treatment-decisions. Participants connected to life differently through personally meaningful activities in line with their own connectedness orientation. The extent to which participants felt connected to life differed. Second, their acceptance of life’s finiteness differed equally. Third, both their connection to life and acceptance of its finiteness colored participants’ overall willingness to be treated and their treatment goals. Fourth, multiple factors were described as disconnecting (physical suffering, unenjoyment food, non-recognition self, living context), while relationality between healthcare provider and receiver was considered connecting. Accordingly, these factors mediated treatment decisions.

**Conclusions:**

Older adults’ overall willingness to be treated and subsequent treatment goals are colored by the extent to which they feel connected to life and accept life’s finiteness. The framework of connectedness to life could provide healthcare professionals with words to elicit and interpret what really matters to older adults during clinical decision-making, and therefore help tailor person-centered care.

**Supplementary Information:**

The online version contains supplementary material available at 10.1186/s12877-026-07736-9.

## Background

Many older patients within the clinical healthcare setting present multiple conditions [1–6], resulting in a complex health status and a large heterogeneity between individual patients. Traditional disease-oriented healthcare, by contrast, builds on relative homogeneity and follows disease-specific guidelines, which guide patients with single conditions towards objectified disease-specific outcomes and, ultimately, optimal survival [1, 2, 5, 7–10]. The discrepancy between disease-based guidelines and older patients’ multi-morbidity creates complex treatment decisions regarding treatment burden, life expectancy, mortality, and patient reported outcomes. In order to navigate these complex clinical care decisions, health care professionals can be guided by older patients’ individual preferences and values, which also display a high level of heterogeneity.

Person-centered care is a viable approach to complex clinical care decisions and capable of bridging the gap between the homogeneous-perspective underlining disease-specific healthcare and the heterogeneous characteristics of older patients [11, 12]. A fundamental aspect of person-centered care is the decision-making process. Healthcare professionals and older patients are guided by diverse frameworks and decision aids during this process, such as shared decision-making [6, 9]. However, numerous studies indicate that healthcare professionals still struggle with eliciting older adults’ subjective preferences and values, and with translating them into disease specific treatment outcomes. Moreover, in the case of an acute disease with constraints in time and communication, discussing older adults’ care needs becomes more challenging [5–7, 9, 11–16].

Eliciting and explicating older adults’ preferences, values, beliefs, and wishes could be facilitated by the holistic framework of vitality through connectedness [17]. A previous study demonstrated how this framework (see Appendix 1 for a figure illustrating the framework) is capable of capturing what really matters to older adults. According to this framework, older adults’ vitality (understood as their zest for life, life-energy, and sense of aliveness) arises from connecting to life itself through internal connectedness (a sense of connection and unity with the self, such as self-continuity, meaningfulness in life, purpose in life, and autonomy) and external connectedness (a sense of connection between the self and the external world, such as social connectedness, and the environmental connectedness with your built and natural surroundings). Every older adult demonstrates a personally preferred way of connecting to life itself. This individual connectedness orientation is defined as an intrinsic inclination towards primarily internal connectedness or external connectedness, or a particular combination of multiple sub forms of both internal and external connectedness [17].

The challenges healthcare professionals experience in clinical decision-making and treatment related goals, raises questions regarding the extent to which older patients’ personal preferences and values guide clinical decision-making [2, 5, 8, 9, 18, 19]. A deeper understanding of what really matters to older adults in life and how this translates into what really matters in clinical decision-making will help deliver person-centered care that is responsive to the heterogeneity within this patient-group. Therefore, this study aimed to explore what really matters to older adults in life and treatment when facing a severe disease, in relation to their connectedness to life.

## Methods

### Research context

The present study was integrated in the COVID-19 Outcomes in Older People (COOP) consortium: a national collaboration in the Netherlands between researchers and healthcare professionals from different care settings, such as hospitals, primary care practices and nursing homes, and a Seniors Advisory Board. Furthermore, the research team of this study followed an interdisciplinary research approach and included multidisciplinary backgrounds such as medicine (WR, VvdK, MM and BvR), general practitioner (JG), internist-geriatricians (FvdB and SM), community medicine and law (YD), sociology (AJ), and health, ageing and society (AJ, WR and VvdK).

### Study design

Our qualitative study followed a thematic content analysis using a directed approach [20–25]. This approach was chosen because the conceptual starting point of the study, and subsequently the initial coding template, was predetermined by the holistic framework of vitality and connectedness [17]. Throughout the analysis however, the directed approach also allowed for the inductive integration of emerging codes into the coding frame and resulting themes [20].

The present study was part of a mixed-method study, which aimed to explore the preferences of older adults’ patient-related health outcomes in case of an acute and/or severe disease, and in relation to frailty status, connectedness and communication. The mixed-method study’s outline comprised a quantitative part and a qualitative part. The quantitative part consisted of a survey-study [16] and the qualitative part consisted of two interview-studies. One of these interview-studies focused on treatment goals [26], while the other (the current interview-study) focused on connectedness. The quantitative survey-study was distributed from May up to October 2022, and included 1278 participants aged 70 years and older, who were community dwelling or living in supportive care facilities in the Netherlands. In this survey, 496 participants indicated a willingness to participate in the qualitative interview-studies. Following purposive sampling based on a wide variety of characteristics (such as age, sex, education, living context, migrant background, experienced health problems, and self-reported Clinical Frailty Scale (CFS) score with four classifications: fit (CFS 1–3), mildly frail (CFS 4–5), severely frail (CFS 6–8), and terminally ill (CFS 9) [16, 26]), 42 participants finally participated in the interview-studies. The study was approved by the Institutional Review Board of the Leiden University Medical Center (LUMC) for observational COVID-19 studies (2022-005). The study was conducted in accordance with the Declaration of Helsinki.

### Data collection and sampling

Forty-two semi-structured interviews were conducted in person between July and December 2022 by four members of the research team with diverse backgrounds (AJ, MM, VvdK and BvR). The majority of the interviews took place at the participants’ residences, and three interviews were conducted at a hospital or rehabilitation and community centers.

One week prior to the scheduled interviews, the recruited persons received an information letter outlining the research aim, procedure, confidentiality, and contact details, and an informed consent form. The interviewers read the informed consent form out loud at the start of the interview and informed consent was obtained verbally. Conform the approved study protocol (Institutional Review Board of the LUMC for observational COVID-19 studies (2022-005)), the audio recorded informed consent was stored digitally to guarantee pseudonymization, confidentiality and an efficient workflow. Pseudonymization of participants and confidentiality of interview data were assured throughout the research project. Further, all semi-structured interviews followed a topic-list, which was pilot tested with the Seniors Advisory Board before data collection started [26] and iteratively revised during data collection based on weekly discussion sessions with the research team. The topic-list covered the topics of connectedness to life, life goals, treatment goals, and communication of treatment goals, including questions such as, “Can you describe what activities are valuable -for you personally- at this time in life?”; “What is important for your future life: which goals, wishes and desires do you have?”; “Which treatment goals and outcomes are important for you if you were to face, or were important to you when you faced an (acute) severe disease?”; “What are your experiences regarding talking about your treatment goals and wishes with others?” (see Appendix 2 for a concise version of the final topic list). Participants were probed about acute and/or severe diseases, experienced by themselves or their loved ones in the past, or currently in the present, or hypothetically in the future. The interviews were audiotaped with participant’s permission and transcribed verbatim by a professional transcription service, after which the researchers manually checked them for anonymity and accuracy.

The 42 interviews served as a pool for the current interview-study and was purposively sampled in order to achieve an equal spread of the characteristics age, sex, education and frailty score, and to include participants with a migrant background and those living in supportive care facilities. Data saturation was achieved after the analysis of 25 interviews.

### Data analysis

The analysis was primarily conducted by two members of the research team (AJ and YD) in close collaboration and dialogue with one other member of the research team (WR) through weekly dialogues in which evolving insights and interpretations were critically discussed from a multidisciplinary perspective. Analytical insights were further discussed during two science meetings, attended by multidisciplinary researchers and clinicians from the LUMC Center for Health and Aging (LUCHA), including the professional disciplines of internists, internist-geriatricians, oncologists, (academic) nurse specialists, general practitioners, community physicians, clinical neuropsychologists, medical biologists, clinical epidemiologists, and sociologists. Moreover, our preliminary results were discussed during a briefing session with a reference group (the COOP Seniors Advisory Board), and their reflections were integrated into the final analysis (see Appendix 3 for an overview of the patient and public involvement (PPI)). Rigor was further supported by maintaining a reflective research journal and reflective memoing, thus ensuring theoretical sensitivity and ongoing reflexivity, transparency and openness [23].

The first step in the analysis was to obtain a notion of the initial sample of transcripts by thoroughly listening to the audio recordings, reading the transcripts and making notes in the margin [23, 25]. During the first phase of data analysis, several overarching concepts of the framework on vitality through connectedness [17] were translated into an initial coding template [20, 23, 25]. The first author (AJ) coded all relevant segments of raw data with the software program Atlas.ti 8 while extending the coding template with emerging inductive codes [20, 23, 25]. Consensual coding was achieved by double-coding (AJ and WR) ten transcripts. Any ambiguous or conflicting codes were carefully discussed and resolved by the research team. Throughout the analysis, more inductively derived codes were added to the evolving coding frame, whereas others were refined or integrated into conceptually congruent categories (axial coding) [23]. Codes and categories were judged on the basis of internal homogeneity (consistency of data within a category) and external heterogeneity (distinctive differences between categories) [25]. During the last phase of the data analysis, categories and themes were grouped further on a conceptual base into overarching themes (selective coding) [23]. Data analysis was repeated while including participants with a broad variety of demographic characteristics, until no new or contrasting insights could be identified in the data. After 22 interviews, the research team discussed data saturation. In order to assure data saturation was achieved, three more interviews were analyzed. As these additional interviews confirmed our insights without elucidating new or contrasting insights, data saturation was considered achieved after 25 interviews [23].

## Results

### Participants

The final sample of this study (*N* = 25) comprised fourteen females and eleven males, with a mean age of 80 years (range, 70–99 years). Nine participants had lower or middle level education and seven considered religion/spirituality important. Fifteen lived alone and four participants lived in supportive care facilities (one assisted living and three nursing homes). Through the self-reported Clinical Frailty Scale (CFS), six participants were classified as fit (CFS 1–3), ten as mildly frail (CFS 4–5), seven as severely frail (CFS 6–8), and two as terminally ill (CFS 9). The demographic characteristics of the participants are outlined in Table 1. The mean length of the interviews was 83 min (range, 34–112 min).

**Table 1:** Characteristics of final sample participants (*n* = 25)

Participant	Age	Sex	Country of birth	Education^a^	Religion/Spirituality	Frailty status^b^	Living context	Residency
P1	81	F	Australia	Higher	Not (very) important	Severely frail	Alone	Independent
P2	85	M	The Netherlands	Higher	Not (very) important	Fit	Together	Independent
P3	79	M	The Netherlands	Higher	Not (very) important	Fit	Together	Independent
P4	81	M	Curaçao	Higher	Not (very) important	Fit	Together	Independent
P5	70	F	Indonesia	Middle	Not (very) important	Fit	Alone	Independent
P6	90	M	The Netherlands	Middle	Important	Severely frail	Alone	Independent
P7	76	M	The Netherlands	Higher	Not (very) important	Mildly frail	Together	Independent
P8	84	F	The Netherlands	Higher	Not (very) important	Mildly frail	Alone	Independent
P9	86	F	The Netherlands	Middle	Not (very) important	Severely frail	Alone	Assisted living
P10	78	F	The Netherlands	Higher	Not (very) important	Severely frail	Alone	Independent
P11	75	M	Surinam	Lower	Not (very) important	Severely frail	Alone	Nursing home
P12	70	F	The Netherlands	Lower	Not (very) important	Severely frail	Alone	Nursing home
P13	73	F	The Netherlands	Higher	Not (very) important	Fit	Alone	Independent
P14	79	F	The Netherlands	Higher	Important	Fit	Alone	Independent
P15	82	F	The Netherlands	Middle	Important	Mildly frail	Alone	Independent
P16	76	M	The Netherlands	Higher	Not (very) important	Mildly frail	Together	Independent
P17	77	M	The Netherlands	Middle	Not (very) important	Mildly frail	Together	Independent
P18	76	F	The Netherlands	Higher	Not (very) important	Mildly frail	Alone	Independent
P19	83	F	The Netherlands	Higher	Not (very) important	Mildly frail	Alone	Independent
P20	78	F	The Netherlands	Lower	Important	Mildly frail	Together	Independent
P21	82	M	The Netherlands	Higher	Important	Mildly frail	Together	Independent
P22	70	M	The Netherlands	Higher	Important	Terminally ill	Together	Independent
P23	81	M	The Netherlands	Higher	Rather not answer	Terminally ill	Together	Independent
P24	82	F	The Netherlands	Higher	Not (very) important	Mildly frail	Alone	Independent
P25	82	M	The Netherlands	Lower	Important	Severely frail	Alone	Nursing home

^a^Education was defined as the highest completed level of education according to the Dutch Verhage scale: lower = primary (vocational) education, middle = secondary (vocational) education, and higher = higher vocational education or university [27]. ^b^Frailty status was defined according to a self-reported Clinical Frailty Scale (CFS): from fit (CFS 1–3) to mildly frail (CFS 4–5), severely frail (CFS 6–8), and terminally ill (CFS 9) [16]

### Thematic description

The following section presents a thematic description of what matters to older adults in life and treatment decisions in relation to connectedness to life. Four main themes were identified following a thematic content analysis. Theme 1 explores the importance of connectedness to life for older adults when facing a severe disease. Theme 2 explores the importance of older adults’ attitude towards the finiteness of life when facing a severe disease. Following from themes 1 and 2, theme 3 describes how treatment goals were colored and defined by both the extent to which participants felt connected to life or not, and the extent to which they accepted the finiteness of life or not. Lastly, theme 4 explores several factors related to disease and treatment that strongly impacted participants’ sense of connectedness to life both negatively (disconnectors) and positively (connector), and accordingly mediated their treatment goals and decisions.

### Theme 1. Feeling connected to life: pivotal for older adults facing a severe disease

When asked about what matters in life when facing a severe disease, participants indicated the pivotal importance of feeling connected to life, and more explicitly, staying connected to their life. Feeling connected to life seemed a general driving force in life, as it strongly related to activities and practices that gave participants life energy, positive affect and a zest for life. Interestingly, when talking about disease and treatment the importance of connectedness to life was restated by participants. Facing a severe disease and undergoing treatment were considered rupturing life-events and disrupting life contexts in which a sense of connection to life specifically came under threat both figuratively and literally; holding on to life itself thus became pivotal.

Participants connected to life through -for them personally- meaningful activities and practices, which could be explicit and concrete, as well as subtle and intangible. Some participants, for instance, mentioned having family over to visit [eg P2] or meeting up with fellow residents at the entrance of the nursing home [P12] (social connectedness), riding their bike [eg P13] or electric wheelchair through strips of nature [eg P11] (environmental connectedness, intrinsic stimuli, independence), making [eg P5] or listening to music [eg P6] and doing hand crafts [eg P5] (self-continuity, intrinsic stimuli), while others mentioned cleaning out the house in preparation of their future death [eg 4] (self-continuity, independence) or gathering information on current events [eg P7] (intrinsic stimuli and goals, engagement). One participant described her meaningful activities as follows:

*“It’s just connecting*,* communicating… with people. But that happens naturally*,* so it doesn’t really matter whether I’m at the shoe repair shop or bump into someone on the street. Yes*,* I basically enjoy having conversations*,* and then I like it when they’re actually about something meaningful. To be honest*,* I just want to ask people: what do you find important in your life? Something like that.”* (P14)

The meaningful activities and practices described by participants illustrated their individual connectedness orientation, and percolated directly into their life goals. For instance, one participant relayed how providing language lessons to migrants was important for feeling connected to life, and one of her life goals was to keep on facilitating these lessons (P18).

The extent to which participants felt connected to life however differed from person to person and could therefore be characterized as a spectrum rather than a dualistic property (e.g. disconnected from life versus connected to life). Their sense of connectedness to life thus ranged from disconnected from life to connected to life, and appeared to be independent of their CFS score. Some participants felt *highly disconnected from life*. They were not able to connect to their life as they had been able in their past life, predominantly due to general ageing. Participants referred to their physical decline, the passing away of loved ones, and the impact of ageism.

*“[I] used to do volunteer work at the art museum here. I had to stop at age 80*,* even though I really didn’t want to let go of that. […] I think it’s a lousy rule*,* because there are people who are already burned out by age 60 and can’t perform anymore. And well*,* I still have it. I’m good at reading people when they come in […] and I can have a lot of fun with people […] Yeah*,* I’m really good with people […] And then you start missing them*,* don’t you? Because I really poured myself into it*,* and that’s a big transition when you’ve been working since you were twenty. […] When all that disappears*,* it’s a really crazy experience*,* because I had so many connections because of it. And then you’re sitting here. And I think that’s awful […] because you identify with that job […]. Yeah*,* I find that a difficult phase*,* when you have to accept that you don’t belong anymore*,* or that you don’t matter anymore*,* or something like that—I don’t know—but that’s really a horrible experience.” (P19)*

Numerous participants felt *relatively connected to life* and were able to engage in a variety of personally meaningful activities. Others felt *highly connected to life* and were able to consciously orchestrate their lives around meaningful activities and practices consistent with their connectedness orientation. Also, they were able to compensate and overcome losses in connectedness successfully by consciously choosing alternative meaningful activities and practices.

“*I am someone who is always looking for solutions. […] So*,* always thinking about: what is still possible? Now*,* with photography for example*,* I quickly adapted myself—I like to photograph small things—to also do it here at home. In the garden*,* for example. I can then sit down and put something I find on the table*,* look at how the shadows fall*,* and then decide whether to leave it there or put it on another table.*” (P10)

### Theme 2. Attitude towards finiteness of life

When asked about treatment goals in relation to facing a severe disease, participants indicated the relevance of their attitude towards a fundamental form of disconnection from life; death, or the finiteness of life. Participants’ attitudes towards life’s finiteness differed from person to person, and could therefore again be characterized as a spectrum, ranging from defying to accepting the finiteness of life. Interestingly, their attitude towards life’s finiteness seemed independent of their current level of frailty. Numerous participants expressed a *clear acceptance of the finiteness of life*. They stressed how they experienced their present life as the tail end of life. These participants emphasized the natural cycle of life, including its inevitable ending. They voiced a conscious consent to the slow process of detaching and disconnecting from life. Besides accepting their future decrease in connectedness to life, this consent also inherently implied accepting a decrease in connectedness to life compared to their past life. These participants could be characterized as being very ‘present’ in their present life. Religion and spirituality were frequently mentioned in this respect, and were seen to reaffirm and soothe the slow process of disconnecting from life. Also, numerous participants mentioned how life became ‘narrow’ or ‘slowed down’. They experienced a sense of ‘emotion amputation’, dampening the depth of feelings ranging from joy and sadness to anger. They did not experience the process of disconnecting from life as bad, and they did not feel this process needed to be defied. On the contrary, it was reflected upon as being a natural part of life; there was no sense in fighting something that natural.

*“I’ve already had my share. Everything I have is a bonus*,* and at some point it will end for everyone*,* for you and for me*,* for everyone. And why should I*,* with all my might*,* try to prolong that?”* (P3)

Participants were also seen to *accept the finiteness of life in a more unconscious manner*. Their acceptance seemed to follow from the slow and subtle lived experience of the natural cycle of life. Other participants were seen to *defy life’s finiteness*. They rejected the slow process of detaching and disconnecting from life. For these participants, this process was not natural, nor an integral aspect of the natural cycle of life. Rather, they expressed a profound and continuous need to feel connected to life. This need, in turn, translated into a strong drive to keep trying to connect to life, a vivid zest for life even. One participant, with a severely frail status, mentioned:

*“I don’t tidy up. Until recently*,* I actually didn’t want to tidy up*,* because it’s a way of preparing for my death. I don’t want that—I want to live*,* thank you very much. […] I have a living will that says: no euthanasia! […] [The doctors] always say: do you want us to resuscitate you? Do you want CPR?” And I said: absolutely.” This was despite the recommendation of my former pulmonologist. He said: yes*,* but do you realize that you’ll never come out of it as well as you went in? And with your condition*,* you’ll end up with broken legs too.” (P1)*

Despite knowingly entering the last phase of life, this knowledge was not so much part of their day to day lived experience.

*“That—at least*,* that’s what I think—I can’t imagine it [death]; you just can’t. […] you just can’t—you can’t imagine it. […] So the best thing to do is just try to enjoy life as much as possible. […] No*,* I don’t really think about it*,* no.” (P20)*

### Theme 3. Overall willingness to be treated and treatment goals

**Fig. 1:**
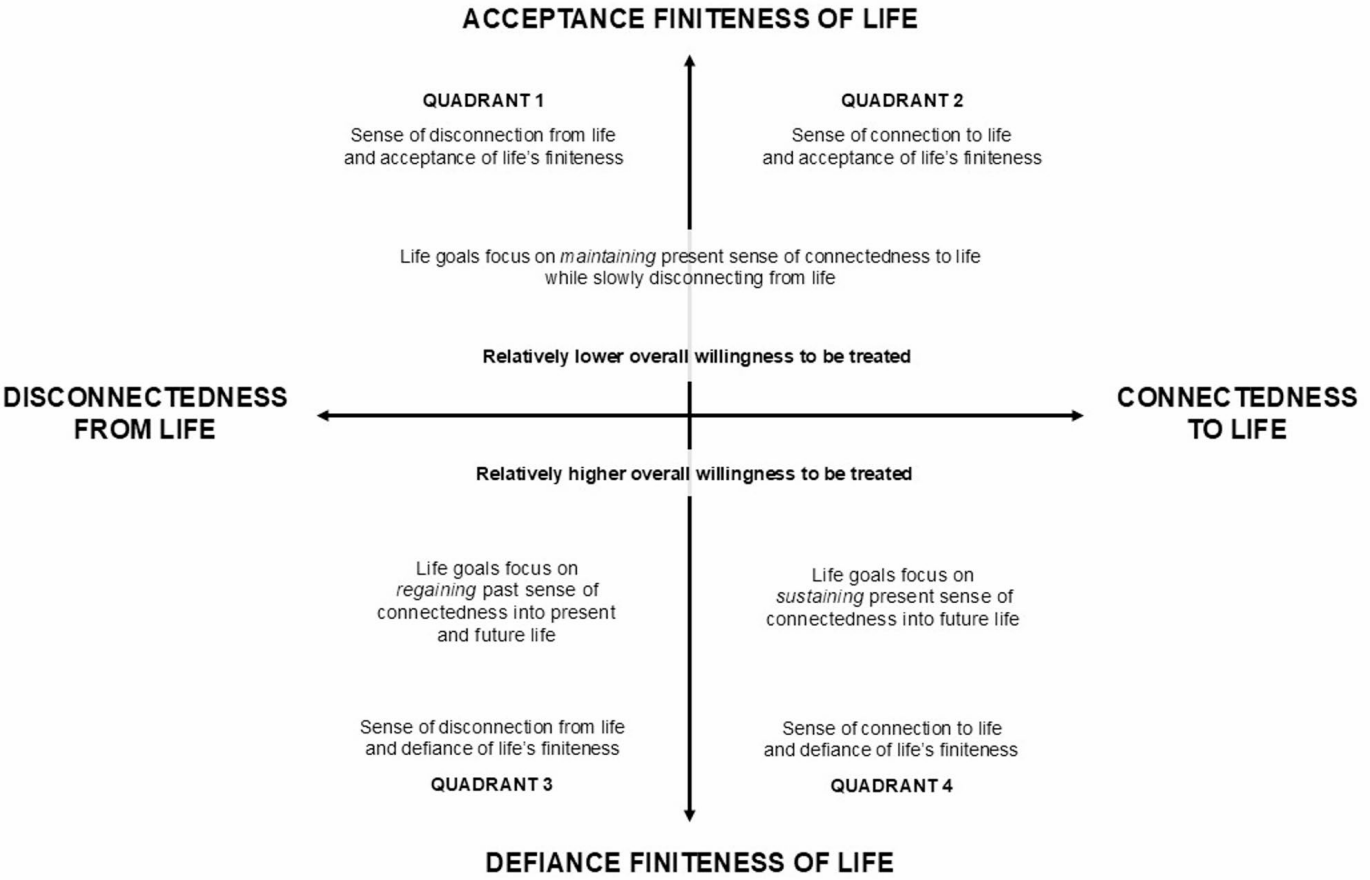
Interrelation between connectedness to life and attitude towards the finiteness of life, which mediated participants’ treatment goals by indicating their willingness to be treated

Following from theme 1 and 2, our findings also indicate that the extent to which participants felt connected to life and the extent to which they accepted the finiteness of life were interrelated, and mediated participants’ treatment goals by indicating their overall willingness to be treated. We illustrate these findings in the axes (axis 1: connectedness to life, and axis 2: attitude towards finiteness of life) and quadrants of Fig. 1.

*Quadrant 1* shows participants who felt fairly to strongly *disconnected from life*, often due to the cumulative impact of ageing, which was experienced as the natural cycle of life. Concurrently, these participants *accepted life’s finiteness*, either unconsciously or through an active and conscious contemplative process. These participants focused primarily on their present life, and their life goals were reflected by maintaining their present sense of connectedness to life, while accepting the slow and steady process of disconnecting from life. They were less adamant regarding prolonging their life in case of a severe disease, regardless of their varying CFS scores. Regarding treatment goals, these participants’ overall *willingness to be treated* was consequently lower. One participant stated how he felt ‘his time had come to an end’, and when asked how this experience impacted him, he answered:

“*That I want to leave*,* I want to die. I won’t commit suicide*,* it’s not that bad*,* but I just don’t feel the need to be here anymore. That*,* I think*,* is it. I’m no longer attached to life. That’s not tragic. That’s how it should be. The cup is full.” : “I feel that euthanasia*,* actually I’m against euthanasia*,* but I’m also against life prolongation*,* and the latter is perhaps even worse than euthanasia. No*,* I definitely don’t want to prolong life. Oh no*,* no treatments. I’ve actually lived my life*,* the circle is complete.”* (P6)

*Quadrant 2* illustrates participants who felt fairly to strongly *connected to life*, despite their consent towards the process of disconnecting from life, and their *acceptance of life’s finiteness*. They explained how they had experienced everything there was to experience and seen everything there was to see. They had lived a full life, they felt content. Consequently, they focused strongly on their present life, rather than the future. The importance of generativity was stressed frequently. Participants relayed how they had successfully passed down their knowledge and experiences to their children and grandchildren, and they felt reassured these family members did not rely on them any longer.

*“Look*,* everyone around me can calmly live their own lives; they are all old and wise enough to now raise children*,* to take care of their own children. There are no more tasks for me to complete.“ : ”No*,* it’s done. I have no more tasks. My tasks are complete. I have no more tasks at all.”* (P3)

Accordingly, their life goals focused on continuing their present life and the current ‘steady state’, while accepting and adapting to life becoming ‘smaller’ and more ‘ narrow’. One participant illustrated this by describing his goals in life as ‘the daily things of a day’. These participants were less adamant regarding prolonging their life in the case of a severe disease. Interestingly, in relation to treatment goals, their *overall willingness to be treated* was lower despite their strong sense of connectedness to life and regardless of their CFS score. Some participants illustrated how they favored quality of life over quantity of life, which meant they would accept only minimal treatment regardless of the severity of the disease. One participant currently faced a life threatening disease and actively decided against treatment, despite feeling strongly connected to life and physically fit.

*“Then I say*,* I’m just not doing that [receive surgery]. And then you might only have a few more years to live. That’s also great! I’m eighty-one*,* what more could I wish for? Then you don’t go through agonizing suffering*,* where you’re not allowed to do anything*,* you’re not allowed to drink beer anymore*,* and God knows what else; pills and rubbish. No*,* not me. […]” : “I’m not having surgery: I’ll have a few years less to live*,* but I have absolutely no desire for that. I’m at peace with that. […] It’s [life] finite*,* and then the end is in sight and close by. That’s how it is*,* and if you accept that*,* it’s peace-giving. You shouldn’t prolong it with chemo and other crap. That’s no longer necessary at my age. No. That*,* I dare say.”* (P23)

*Quadrant 3* illustrates participants who experienced a relatively strong sense of *disconnection from life* and concurrently showed a *defiance of life’s finiteness*. There was a stark contrast between their current sense of disconnection from life and the sense of connection to life they had experienced in their past life. Their life goals subsequently reflected a strong longing to regain this past sense of connection. When asked to describe their life goals they related back to their past life, despite knowing these goals would be unreachable.

*“Ah*,* my longing is mainly that I long for what I had. Very nostalgic.“ : ”Well yes*,* I basically just long for my old life back.”* (P13)

*“What are my wishes? My wishes are so big*,* so many that I can hardly list them all. Because*,* I would like to go on vacation*,* also visit my family in Suriname. But I can’t do that. I’m not allowed to fly. […] Those are my wishes*,* really. But*,* well*,* I know it’s not possible.”* (P11)

In line with their strong longing to regain their past sense of connection, and despite their current sense of disconnection from life, these participants proclaimed a vivid zest for life, a drive and longing to prolong life. Regarding treatment goals, they subsequently shared an intriguingly strong *overall willingness to be treated*, independent of their varying CFS scores.

“*I don’t have what some people have*,* that: oh*,* if I get dementia*,* then I want to end my life right away. No*,* absolutely not*,* because life is sacred to me.”* (P13)

*Quadrant 4* illustrates participants who experienced a relatively strong sense of *connection to life* as well as a clear *defiance of life’s finiteness*. These participants shared a strong longing to maintain their present sense of connection to life, and prolong it into their future life. Their life goals clearly focused on their future life, on pursuing meaningful activities that reflected their connectedness orientation into the future. Participants mentioned how they wanted to keep up nature photography, engage with other people and inspire them, visit museums. Accordingly, in relation to treatment goals, these participants showed a strong *overall willingness to be treated*, independent of their varying CFS scores.

*“I am really very attached to life. I always tell people around me that I want to live to be 100. So I think I would be quick to opt for an unpleasant treatment. […] I’m not really afraid of pain or anything like that*,* or of an unpleasant treatment. I just tell myself: oh well*,* it will pass. I have seen people around me who have had cancer and undergone chemotherapy. I have seen how nasty that is. But then again*,* she did get through it. So I’m not afraid of it either.”* (P10)

### Theme 4. Disease and treatment related factors mediating treatment decisions

#### Disease and treatment related ‘disconnectors’

Participants highlighted four key factors related to disease and treatment that fundamentally impacted their sense of connectedness to life, even creating a strong sense of disconnection from life (disconnectors). These factors were seen to directly impede participants’ life goals and therefore mediate their clinical treatment decisions and overall willingness to undergo (certain aspects of) treatment. These four key factors are:

##### Physical suffering

Numerous participants mentioned how physical suffering resulting from treatment would deter them from choosing for treatment. ‘Pain’ was frequently referred to. Experiencing high levels of pain on a daily base made it impossible for participants to connect internally or externally. For instance, pain could prevent them from engaging in meaningful activities that sparked their intrinsic curiosity, or helped them experience a goal in life, or independence (internal connectedness). Also, pain could hinder them in activities as meeting friends, gardening, or reading about current affairs (external connectedness). The experience of pain could be so overwhelming and all-consuming that it could even disconnect them from their own thoughts and their sense of self (internal connectedness). Moreover, pain medication was feared for exactly this reason, as the side effects could make them numb and ‘groggy’.

*“That’s what I noticed when I had cancer and was given Oxycodone and Fentanyl. Yes*,* it makes you as drowsy as a monkey. Half the time I fell asleep. Well*,* then they might as well just put me to sleep for good*,* yes*,* and then that’s it*,* bye-bye.”* (P3)

Some participants currently experienced chronic pain but despite this had learnt how to stay connected internally with their self through extensive rehabilitation therapy focused on mindfulness. Strikingly, for them, physical suffering was no longer a factor mediating treatment goals.

##### Inability to enjoy food

Several participants spoke about the impact of losing their ability to enjoy food. In some cases this inability was context based, for instance when participants were not able to eat according to their own preferences within a nursing home, which fed into their resistance towards nursing homes and impacted treatment goals accordingly. More often, however, the inability to enjoy food was physical, due to a severe disease or a related treatment. For these participants the impact was more severe, draining their zest for life and their life-energy both literally and figuratively. For several participants, losing their ability to enjoy food created such an existential sense of disconnection that they lost their willingness to be treated, and to prolong life. As a result, these participants were seen to actively contemplate ending their life.

##### Non-recognition of own appearance

Some participants mentioned the impact of not being able to recognize their own appearance, for instance due to changes in their physical appearance resulting from severe disease or treatment. Not recognizing their own appearance created a strong disconnection from their identity and personhood, and arrested their sense of self-continuity (internal connectedness), which could lead to questioning the worth of their present life and, accordingly, their willingness to prolong life and proceed with treatment. One participant shared how after a childhood plagued with disease and infirmity, he had been a heavy but strong man throughout his adult life. These physical attributes held an important symbolic value closely related to his internal and external connectedness. His strength symbolized being trustworthy and safe, an ability to help others, and accordingly his sense of mission and self-worth. Subsequently, he experienced the loss of these physical attributes as a loss of connection to his self and others.

*“I also told my wife: if I had known that*,* I wouldn’t have had it done [life-saving surgery]. It sounds a bit strange*,* but my quality of life has changed so much. I’m not used to it. […] I’m not a macho type*,* but I did used to be nice and strong*,* I was always good and fit. And then*,* all of a sudden*,* I went from 120 to 72 kilos.”* (P22)

In contrast to actual changes in physical appearance, other participants pointed towards changes in cognitive functioning, such as dementia, creating an inability to recognize oneself. For these participants, not being able to recognize yourself meant being disconnected from the self. They underlined the notion of being physically connected to life while being mentally disconnected from life, which equaled a loss of self. Accordingly, they would not want to prolong their life and be treated.

*“I need to retain my mental faculties—well*,* my mental abilities*,* that is. If I can’t do that anymore*,* if I*,* let’s say*,* become as demented as a door*,* and my wife comes to visit me*,* and at that moment I ask the nurse*,* “Who is that lady?” To which she says*,* “That’s your husband*,* that’s your partner.” And I say: “Oh*,* mm-hmm.” Or my son comes in. If I can no longer recognize them*,* then I’m a plant. Then I am—and I don’t want to live like a plant. That*,* sorry*,* I don’t want that.” (P4)*

Contrastingly, one participant mentioned in case of dementia she would not mind moving to a nursing home exactly because of the presumed loss of self:

*“Unless I develop dementia*,* then I’m fine with it*,* because then I won’t remember anything anyway. That’s a completely different issue. […] So in that sense*,* if I develop dementia*,* they can just put me anywhere. I won’t know anymore anyway.”* (P13)

##### Living context

Numerous participants were adamant regarding not moving to a nursing home, and were willing to adjust their treatment goals accordingly. Some mentioned how they feared they could no longer ‘live their life’ or ‘be who they are’ in a nursing home, creating a loss of internal and external connectedness. For instance, participants mentioned they would no longer be able to walk through their neighborhood (external connectedness), follow their own daily routine (independence), engage in activities and practices following their own preferences (internal connectedness), or feel mirrored by other nursing home residents (external connectedness).

*“Well*,* I don’t think I’m really into big groups. I mean*,* there are 63 people in a place like that. […] and everyone there is suffering from something. Well*,* then the conversation was always about what you were suffering from. And there were a lot of gardeners*,* and I mean*,* they’re nice people*,* […] but that’s what the conversation always ended up being about. So yes*,* I didn’t find anyone for a little chat*,* but I also didn’t find anyone who made me think*,* “Gosh*,* I’m going to sit at the table with them for a while*,* just to have a nice time.”* (P18)

Accordingly, participants stressed the importance of their own home, highlighting how the furnishings and decorations were a constant reminder of who they are (self-continuity), or even a direct extension of the self, helping them connect internally to their self.

*“That’s the beginning of the end [a nursing home]. The moment you’re taken away from your environment*,* you lose your entire past. You can’t see it anymore. They take some photos with them […] and that’s all they have to go on. […] Look*,* this is all on offer. Whether I ask for it or not*,* it’s there. With most of the objects here*,* I can picture the story behind them.”* (P21)

The feared loss of self when residing in a nursing home is reinforced by our earlier finding regarding participants’ willingness to move to a nursing home when facing a form of dementia, as they ‘would no longer be themselves’, or be aware of their self. One participant would accept moving to a nursing home in the future as long as she could stay true to who she was.

*“So if it becomes no longer possible*,* I have already given the people around me permission to simply send me to a nursing home. Yes*,* but I did write down how I would then like it to be. What I find important. Yes*,* that it’s a place where I can feel at home: where there are beautiful things*,* where I can wear the clothes that are part of who I am. Not that I’m so special or anything*,* but something that suits who I am*,* that my hair is cut by a hairdresser who does it the way I am now. Yes*,* and that there are nice people and that I don’t have to play bingo. Maybe something with nice music.”* (P10)

Interestingly, residing in a hospital or rehabilitation center for treatment generated similar feelings of disconnectedness, and mediated participants’ willingness to undergo treatment, or the length of treatment they were willing to undergo. For instance, one participant’s sense of connectedness to life relied mainly on her casually meeting up with fellow nursing home residents (external connectedness). Regarding a possible hospital admission due to severe pneumonia, she said:

*“Because with that lung infection they started like: if it doesn’t get better*,* you’ll have to go to the hospital. I said: well*,* no hospital for me. I’m just staying where I am. I no way feel like going to a hospital. If I have to*,* I have to. Yes*,* but what I thought was: I want to do everything I can to just stay here. […] otherwise you’re just lying there all alone*,* and no*,* I didn’t feel like that at all.”* (P12)

#### Disease and treatment related ‘connectors’

On the other hand, a key factor impacting participants’ sense of connectedness to life positively (connector) during treatment was relationality between healthcare provider and receiver. Relationality can be considered as an equivalent of social connectedness, which is a sub form of external connectedness. However, besides facilitating external connectedness, relationality with a healthcare provider simultaneously fostered participants’ internal connectedness by cultivating an environment in which older adults could share information regarding their own life, intrinsic stimuli and goals, their independence. Subsequently, relationality with a healthcare provider enabled participants to connect internally, despite entering a disrupting life context (clinical setting) and facing a disrupting life-event (severe disease). Three key aspects of relationality between older patients and healthcare professionals during treatment were derived inductively, adding to our understanding of connectedness beyond the existing framework:

##### Relationality at system level

Overall, participants wished for a holistic healthcare approach in which their entire being was heard: physical, mental and spiritual. The ability to talk about all these dimensions within a clinical setting enabled older adults to feel a deeper sense of social connectedness with healthcare professionals. Concurrently, being able to convey what mattered to them mentally and spiritually fostered participants in connecting to their own life.

Participants mentioned healthcare professionals’ focus on the body, specifically on certain parts of the body. However, participants frequently experienced subjective changes throughout their body as well as their mental and spiritual well-being when facing a medical problem with a specific part of their body. They explicated how they felt no ‘room’ nor time to consult their medical specialist about this general impact, or ask related questions. The fragmented approach of healthcare created a sense of confusion and disconnection in understanding and feeling their own body properly, decreasing internal connectedness.

*“Because I don’t get answers to questions I have in my head*,* questions I want to ask but don’t dare to because I think: ‘Maybe I’m being too much.’ And: ‘It’s only fifteen minutes*,* I can’t stay here any longer because they only have fifteen minutes for me’. But I would so much like to just attend a consultation at the hospital and have them say: ‘I’m the oncologist and let’s sit down for a moment and take our time. We’re not watching the clock*,* so ask me any questions you have’. And then I’ll try to explain it as best I can.”* (P22)

##### Relationality at interpersonal level

Participants considered a sense of ‘humanity’ valuable in the way healthcare professionals treated patients. They wished healthcare providers and receivers could relate to each other as human to human. For instance, participants wanted their distinctive personhood to be seen and acknowledged by healthcare professionals, cultivating a deeper sense of social connectedness. The act of being heard and acknowledged in their personhood simultaneously fostered older adults’ internal connectedness by helping them reconnect or stay connected internally despite their disease and throughout treatment.

*“That they have an eye for the whole patient*,* because specific issues stem from somewhere*,* so not just focus on that tiny little part of the person. […] A patient consists of many different things*,* including their own opinion*,* and everything. […] I don’t think a doctor can just determine something like that. Look*,* an X-ray or a scan*,* can point out the issue*,* anyone can see that. However*,* a treatment must suit the patient.”* (P8)

*“I would have loved for him to say: ‘Madam*,* you’ve made it through*,* it’s going well’. Or something like that*,* you know. ‘The danger has passed’. I would have so loved to have heard that. But when I said: ‘Could I still die?’ […] He replied: ‘Well*,* madam*,* we could all die any day’. I thought that was so heartless and stupid. […] I think it’s such a shame that people don’t have more compassion for others. […] I really think it’s very important if someone is a bit human.”* (P20)

Further, participants explicitly described their longing for a loving approach in which they felt being cared for. Interestingly, this longing to feel cared for exceeded healthcare and medical treatment. In contrast, what participants highlighted was an intuitive, attentive, fundamentally human form of ‘caring for’ and social connectedness. They did not feel this would disturb possible professional etiquettes.

##### Relationality at personal level

Participants considered their own responsibility in fostering relationality valuable, and several participants stressed the value of reciprocity. Several participants wished to build and grow a relationship with healthcare professionals and to help healthcare professionals care for them. Healthcare was considered a team effort, and it was a patient’s own responsibility to take part in the team.

*“And I try to take things off their hands too. Little things. When you’re lying in bed*,* you get all kinds of things and food*,* and so on. It just becomes a mess. I have a few large plastic bags*,* so I keep my table a bit clean*,* and when the bag is full*,* I throw it in the trash can*,* and that relieves the staff. They then don’t have to clean up my mess.”* (P7)

*“So*,* that is very important*,* of course*,* that you get acknowledged and that you build a good relationship with someone. And I think it’s very important to maintain that contact. […] Yes*,* I have all those things*,* they’re very important. But*,* I do think that if I want to have a good relationship*,* I have to make an effort myself.”* (P19)

Accordingly, the mutual transfer of knowledge between healthcare professionals and patients was perceived as valuable. Communication depended on willingness, honesty and trust from both healthcare provider and receiver. Creating a mutual understanding and common ground was considered imperative. Some participants mentioned they did not want to be ‘treated as a child’. They wanted to receive all relevant information, including possible distressing details. Other participants however expressed their fear of being considered a burden by taking up too much time and attention in healthcare professionals’ busy schedules. This fear hindered them in taking responsibility for building relationships and acquiring knowledge.

*“Because I often feel*,* even at the hospital with that oncologist who knows me very well*,* that I can’t completely express myself properly. I don’t dare to*,* because I think she doesn’t have time for that. She’s a specialist in this*,* but not really in that. That’s the impression I get.”* (P22)

## Discussion

### Reflections on findings

In this study, we aimed to explore what really matters to older adults in life and treatment in relation to connectedness to life [17]. First, our findings revealed that a general driving force in life for participants was feeling internally and externally connected to their lives. They considered this sense of connectedness to become pivotal when facing a severe disease and undergoing treatment. Facing a severe disease was seen as a rupturing life event, and undergoing treatment created a disrupting life context; both of which threatened participants’ connectedness to life indirectly and directly. Our findings showed that participants connected to life through personally meaningful activities in line with their connectedness orientation, even participants with varying CFS scores. The extent to which participants felt connected to life differed, ranging from disconnected from life towards connected to life. Second, when facing a severe disease and considering treatment goals, participants’ attitude towards the finiteness of life appeared to be important, and could range from defying the finiteness of life to accepting it. Third, participants’ sense of connectedness to life and their acceptance of life’s finiteness interrelated. Subsequently, both factors colored and defined participants’ goals of treatment through their overall willingness to be treated, independent of their CFS score. Strikingly, some participants were seen to consciously accept the finiteness of life despite feeling fairly to strongly connected to life, and would consequently show a relatively low overall willingness to be treated. Additionally, other participants were seen to defy life’s finiteness despite feeling fairly to strongly disconnected from life, showcasing a relatively high overall willingness to be treated. Lastly, our findings revealed factors related to disease and treatment that strongly impacted participants’ sense of connectedness to life both negatively (disconnectors) and positively (connectors), and accordingly mediated their treatment goals. While physical suffering, inability to enjoy food, non-recognition of own appearance, and living context were experienced as highly disconnecting, relationality between healthcare provider and receiver was considered highly connecting.

Our results contribute to the existing literature on person-centered healthcare for heterogeneous older adults in multiple ways. To our knowledge, our findings are novel regarding the importance of older adults’ sense of connectedness to life when facing a severe disease and subsequent treatment decisions. For older adults, the need to stay connected to life through personally meaningful activities and practices forms a starting point when defining treatment goals, and thus a first step in the decision making process. Older adults’ heterogeneity should therefore not merely be considered from a somatic or geriatric (CFS) perspective. Instead, our results show the relevance of their heterogeneity in personhood (e.g. their preferences, values and life goals) for clinical decision-making.

Furthermore, our study underlines the relevance of the framework of vitality through connectedness [17] for clinical decision-making as it helps healthcare professionals and older patients explicate what matters in life and treatment. First, this framework might provide relevant words to elicit preferences and values, as it gives expression to what older adults perceive valuable in life through highlighting activities and practices that drive their zest for life and life goals. Second, the framework might help interpret the words older patients (tend to) use during clinical decision-making in a new way, as it creates a deeper understanding of why these activities and practices matter so much by showing how they create connectedness to life, a feeling of aliveness and vitality. Similarly, our study provides insight into why older adults experience specific factors as barriers (e.g. physical suffering, inability to enjoy food, non-recognition of own appearance, living context): these factors form obstacles for older adults’ internal and external connectedness to life. Both points (relevant words and relevant interpretations of words) could help healthcare professionals depart from a person*hood*-centered starting point during clinical decision-making, in addition to the fundamentally clinical starting point of shared decision-making, which aims to elicit preferences, values, and goals that are unambiguously relevant to universal health outcomes [9, 12, 28].

Lastly, our findings regarding the need for relationality in person-centered healthcare are echoed by multiple studies that address a lack of holistic care and loss of a human element in care [1, 26, 29–31]. Studies on integrated care and multidisciplinary teams emphasize that the body should be considered as a whole, including mental well-being, and frequently include patients’ widening relational circles in their focus [29]. Other scholars focus on the relation between religion and spirituality, and health [30]. However, our emphasis on relationality and social connectedness between healthcare provider and receiver provides a more novel perspective. Resnick [32] highlighted this perspective in an editorial, stressing the importance of relating and connecting to a patient beyond ‘task completion, patient education, or the expression of beliefs about what the patient should know and do’. Also, she emphasizes this approach would not be more time-consuming. Other researchers echo this, and indicate how healthcare professionals in acute medical units were able to elicit patients’ preferences and values despite limited available time by asking quick and simple, yet profound questions, such as “what matters to you?”, followed by “why does this matter to you?” [33] Similarly, recent research [34–38] focusing on ‘embodiment’ underlines the positive affect of relationality on both patient and healthcare professional. Rather than understanding care as a cognitive activity in which learnt skills are executed, this field of research emphasizes the lived experience of ‘caring for’, that also entails physical relationality through caring touch (e.g. ‘skin hunger’).

Altogether, our results indicate several implications for clinical decision-making with older patients. A broader understanding of their heterogeneity -one that moves beyond a geriatric frailty perspective, and incorporates their ‘being’ and personhood, their sense of connection to life, and their attitude towards the finiteness of life- might enable healthcare professionals to identify specific groups of older patients, and formulate relevant treatment decisions (personally and clinically). For example, relatively frail older patients with a strong overall willingness to be treated, despite treatment not being medically worthwhile, for whom treatment decisions could include welfare or existential/spiritual guidance to help them connect internally and to life. Or relatively fit older patients who do not want to be treated, despite treatment being medically worthwhile. For these patients, decision making could include shifting the focus to palliative care instead invasive treatment, and prioritizing their sense of connection to their life during the remainder of it. Furthermore, integrating specific words and interpretations from the framework of vitality through connectedness [17] into clinical decision-making does not imply additional time-consuming communication models, merely a shift in usage and understanding of words.

### Strengths and limitations

This study presents several strengths. First, our explorative study-approach gave voice to older adults’ (and thus emic, insider) perspectives on treatment and related goals. In doing so it suggests implications that can serve to enrich and extend the existing clinical (and thus etic, outsider) perspective on clinical decision-making by providing words that can help elicit and explicate what really matters to heterogeneous older adults in line with person-centered care. Second, our multidisciplinary research team facilitated interdisciplinary research by integrating findings in multiple knowledge silo’s, which solidifies the rigor, robustness, and implications of our findings. Also, incorporating other disciplines, in addition to the medical, into the research team facilitated an open and unbiased attitude towards the data, describing emic perspectives of older adults themselves. Third, our study included a highly varied sample of older participants through purposive sampling of the broad pool of participants in our quantitative study, including participants with various educational backgrounds, CFS scores and living arrangements.

Besides strengths, this study exhibits several limitations. Our findings concern both actual and hypothetical decision-making. Participants discussing the latter could possibly have answered differently when facing actual decision-making. Also, this study was conducted in The Netherlands, which is a high-income Western-European country with an advanced and accessible healthcare system, as well as a highly individualized culture. This context possibly influenced the notion of ‘choice’ in relation to treatment goals, and the experience of relationality in health care. The transferability of our findings to older adults in other economic and socio-cultural environments is a relevant topic for further research. Nevertheless, our explorative findings point to fundamental notions that merit further research regarding person-centered care in other high-income countries. Moreover, following the COOP Seniors Advisory Board’s recommendations, which emphasize the importance for older adults of integrating research findings into future healthcare practice, further research should investigate how our findings on older adults’ connectedness to life can be integrated into clinical decision-making.

## Conclusions

Connectedness to life plays a vital role for older adults in life, disease and in treatment decisions. Older adults’ overall willingness to be treated and their subsequent treatment goals are colored and determined by the extent to which they feel connected to life and accept the finiteness of life. These insights illuminate a broader understanding of older adults’ heterogeneity, incorporating their personhood, their values and what they consider valuable in life. During clinical decision-making, healthcare professionals and older patients could be guided by the framework of vitality through connectedness [17], as this framework could provide relevant words to elicit, and interpretations to understand the relevance of what really matters to older adults. Subsequently, it can help tailor person-centered care to this heterogeneous patient group. Future research could further explore how the framework can help align older adults’ preferences and values with clinical practice.

## Supplementary Information

Supplementary Material 1.

## Data Availability

The datasets generated and/or analysed during the current study are not publicly available due to the qualitative nature of these data [the interview transcripts contain attributable sensitive information that potentially identifies participants and would breach participant confidentiality if made publicly available] but are available from the corresponding author on reasonable request. All other relevant data generated or analysed during this study are included in this published article [and its supplementary information files].
