# Supplementary material for: What really matters to older adults in life and treatment: a qualitative study on the role of connectedness in person-centered care
Source: BMC Geriatr. 2026 Jun 3;26:994. doi: 10.1186/s12877-026-07736-9 (PMC13425873; doi:10.1186/s12877-026-07736-9)
Supplement: Supplementary file 1 — Supplementary Material 1. [file 12877_2026_7736_MOESM1_ESM.docx]

**What really matters to older adults in life and treatment: a qualitative study on the role of connectedness in person-centered care**

Anneke G. Julien, Willeke M. Ravensbergen-Roobol, Veerle M. G. T. H. van der Klei, Mabel J.E. Maissan, Bas F. M. van Raaij, Frederiek van den Bos, Simon P. Mooijaart, Jacobijn Gussekloo, Yvonne M. Drewes

**Appendices**

Appendix 1: Figure illustrating the framework of Vitality through internal connectedness and external connectedness [1]

Appendix 2: Concise version of the topic list of the COOP-study on goals of care

Appendix 3: Patient and Public Involvement (PPI), following the GRIPP2 short form [2], in the COOP qualitative substudy on what matters to older adults in life and treatment, in relation to connectedness to life.

| **Appendix 1.** Figure illustrating the framework of Vitality through internal connectedness and external connectedness [1] |
| --- |
| **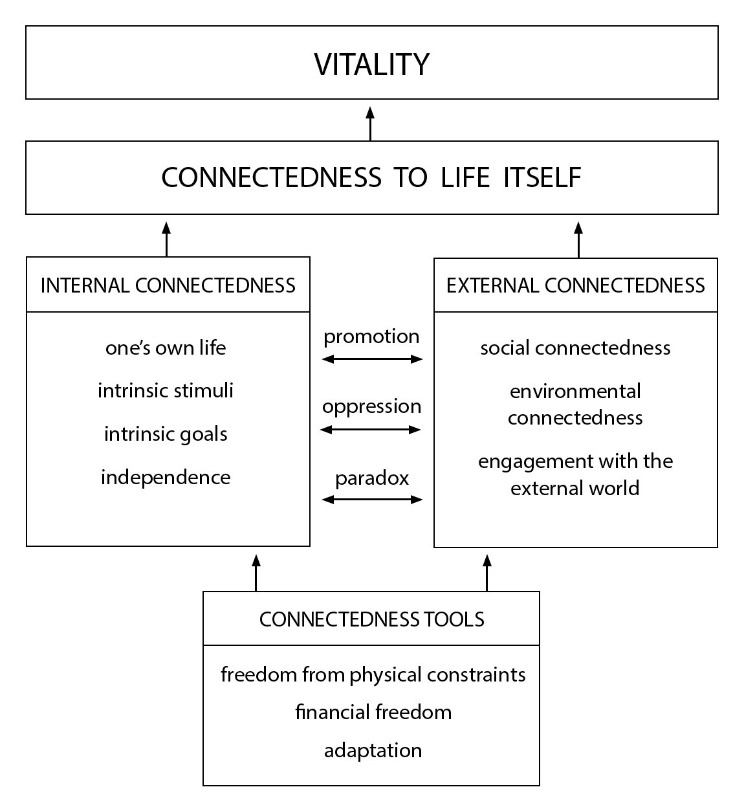** |
| **Figure description** Theoretical, holistic conceptualisation of vitality achieved by feeling connected to life itself, through internal connectedness (constituents: connecting to one's own life, intrinsic stimuli and intrinsic goals in an independent manner) and external connectedness (constituents: social connectedness, environmental connectedness and engagement with the external world). The connectedness tools can facilitate internal and external connectedness, whilst the absence of these tools can function as a barrier. At the intersection of internal connectedness and external connectedness distinctive interactions emerged. |
| **Appendix 1.** Concise version of the topic list of the COOP-study on goals of care (forward-translation). |
| **Topic 1. Valuable activities and life goals**   - Could you describe which activities are currently valuable to you in daily life? - Why are these activities important or what do you attain with these activities? - How did these activities evolve over time? If applicable, after [disease or life event]? - If applicable, what did you need to adapt your valuable activities in the past? - What matters regarding your life in the future? Which goals, wishes or desires do you have? - Out of your valuable activities, what do you preferably want to preserve in the future? - Out of your valuable activities, what do you minimally need for a good quality of life (QoL)? |
| **Topic 2. Goals of care in case of acute and/or severe disease**   - What is your experience with being ill? If applicable, with acute and/or severe disease? - What were important outcomes to you after [your disease and care experience]? - When being ill, “goals of care” are often discussed. How do you define goals of care? - What if you would (again) become acutely or severely ill, what would matter to you regarding your medical care and treatment? Which goals would you have regarding your care and treatment? Or what outcomes should minimally be attained with your care and treatment? - What are you willing to give up in case of care or treatment to preserve your QoL? - “Independence” is often discussed in health care. How do you define (in)dependence? - If applicable, how did you end up living in a nursing home? How did you regard this in the past? |
| **Topic 3. Preferred communication about these goals**   - What is your experience with sharing your goals of care with others? If applicable, with your family, other medical representatives and with (un)known health care professionals? What facilitated or hampered you in sharing these goals? If applicable, what role did others have? - How do you prefer sharing your goals of care in case of future acute and/or severe disease? What if you cannot share your goals yourself (e.g. delirium)? What do you need from others? - In what way are you now considering the chance of becoming acutely and/or severely ill? To what extent did the COVID-19 pandemic influence this awareness? How do you prepare or how should health care professionals be best prepared to care for you in case of a new pandemic? |

| **Appendix 2.** Patient and Public Involvement (PPI), following to the GRIPP2 short form [2], in the COOP qualitative substudy on what matters to older adults in life and treatment, in relation to connectedness to life. | |
| --- | --- |
| **1. Aim**  Report the aim of PPI in the study | To give voice to older people who are ‘experts by experience’ based on their age, diverse medical histories and experience with the COVID-19 pandemic in the Dutch context by collaborating with them in our exploration of what really matters to older adults in life and treatment when facing a severe disease, in relation to connectedness to life. |
| **2. Methods**  Provide a clear description of the methods used for PPI in the study | Ten older people were recruited to participate in the COOP Seniors Advisory Board: median age 75 years old (IQR 70-78), 70% female and 100% higher educated*. They had diverse medical experiences (e.g. history of COVID-19 or other disease and ranging from fit to mildly frail) and professional backgrounds (health care and research, anthropology, education and finances). 70% also represented another senior organization spread across the Netherlands.  Throughout the COOP-project the Board participated according to all five roles of the Involvement-Matrix: listener, co-thinker, advisor, partner and decision-maker [3]. Their chairman was part of the steering committee of the larger COOP-consortium and was co-leader of this substudy on goals of care. He was involved from the very beginning in defining the research question and drafting the grant application. Subsequently, the Board was involved in the study design, data collection and interpretation of the findings of this qualitative substudy. |
| **3. Results**  Outcomes—Report the results of PPI in the study, including both  positive and negative outcomes | The COOP Seniors Advisory Board influenced this substudy as follows:   - The Board repeatedly underlined the relevance of the research question central to this substudy. - The Board improved the heterogeneity of our sampling frame by revising and distributing the preceding quantitative questionnaire. - The Board participated in pilot interviews to train the interviewers and to experience the initial topic list from the lay perspective, after which minor revisions were made to improve its comprehension. - The Board was repeatedly involved during the data analysis in discussions about the (preliminary) themes to complement the professional perspective of the research team. Discussions about connectedness took place during one meeting which was specifically organised to discuss the emerging findings of this substudy. - The chairman of the Board shared reflections on the manuscript (e.g. suggesting follow-up research on translating the results of this substudy into clinical practice), and suggestions were accordingly incorporated in the article.   For pragmatic reasons, the Board was not directly involved in the further analysis of this substudy, as this analysis took place mostly after rounding off the formal meetings of the Board. The board experienced this as a missed opportunity, seen the relevance they subscribed to this substudy. |
| **4. Discussion and**  **Conclusions**  Outcomes—Comment on the extent to which PPI influenced the study  overall. Describe positive and negative effects | PPI involvement of older people in the COOP study encompassed varying roles of the Involvement-Matrix throughout all research stages. For this substudy, it enhanced the study’s inclusiveness for the heterogenous older population. Furthermore, the discussion of the emerging themes with the Board, as well as the final reflections by the Board’s chairman on the manuscript improved the relevance of our results |
| **5. Reflections,**  **critical perspective**  Comment critically on the study, reflecting on the things that went well  and those that did not, so others can learn from this experience | The involvement of the Seniors Advisory Board in the COOP study was overall very positive. The Board was rapidly established via the large network of the COOP-consortium. The extensive previous experience of the chairman and senior researchers with PPI, together with professional training in PPI for the junior researcher involved, facilitated fruitful collaboration throughout the COOP project. This substudy benefited greatly from the rich sampling frame, which was collected within the COOP project and served as pool for this substudy.  As the Board was not directly involved in the further analysis of this substudy, communication regarding developments arising in diverging timelines and in the continuation of participation is advisable. |
| **Notes**: ^*^According to the Dutch Verhage Scale on educational attainment.  **Abbreviations**: COOP, COVID-19 Outcomes in Older People consortium; GRIPP2, Guidance for Reporting Involvement of Patients and the Public version 2; IQR, interquartile range. | |

1. Julien, A. G., den Elzen, W. P. J., Reis, P. R., Touwen, D. P., Gussekloo, J., & Drewes, Y. M. (2024). Vitality of older adults through internal and external connectedness. *Aging and Health Research*, 100185. https://doi.org/https://doi.org/10.1016/j.ahr.2024.100185
2. Staniszewska S, Brett J, Simera I, et al.; GRIPP2 reporting checklists: tools to improve reporting of patient and public involvement in research. *BMJ* 2017;**358**:j3453. doi: 10.1136/bmj.j3453.
3. Smits DW, van Meeteren K, Klem M, et al.; Designing a tool to support patient and public involvement in research projects: the Involvement Matrix. *Res Involv Engagem* 2020;**6**:30. doi: 10.1186/s40900-020-00188-4.

**REFERENCES**
